# Design and Implementation of Real-Time Localization System (RTLS) Based on UWB and TDoA Algorithm

**DOI:** 10.3390/s22124353

**Published:** 2022-06-08

**Authors:** Fengyun Zhang, Li Yang, Yuhuan Liu, Yulong Ding, Shuang-Hua Yang, Hao Li

**Affiliations:** 1 Shenzhen Key Laboratory of Safety and Security for Next Generation of Industrial Internet, Southern University of Science and Technology, Shenzhen 518055, China; zhangfy2019@mail.sustech.edu.cn (F.Z.); liuyh2019@mail.sustech.edu.cn (Y.L.); dingyl@sustech.edu.cn (Y.D.); yangsh@sustech.edu.cn (S.-H.Y.); 2 Department of Computer Science and Engineering, Southern University of Science and Technology, Shenzhen 518055, China; 3 Army Engineering University of PLA, Nanjing 210000, China; yangli.aeup@gmail.com; 4 Academy for Advanced Interdisciplinary Studies, Southern University of Science and Technology, Shenzhen 518055, China; 5 Science and Technology on Near-Surface Detection Laboratory, Wuxi 214000, China

**Keywords:** indoor localization, ultra wide-band (UWB), time difference of arrival (TDoA), wireless clock synchronization (WCS), time-base selection strategy, extended Kalman filter (EKF)

## Abstract

Nowadays, accurate localization plays an essential role in many fields, such as target tracking and path planning. The challenges of indoor localization include inadequate localization accuracy, unreasonable anchor deployment in complex scenarios, lack of stability, and the high cost. So, the universal positioning technologies cannot meet the real application requirements scarcely. To overcome these shortcomings, a comprehensive ultra wide-band (UWB)-based real-time localization system (RTLS) is presented in this paper. We introduce the architecture of a real-time localization system, then propose a new wireless clock synchronization (WCS) scheme, and finally discuss the time difference of arrival (TDoA) algorithm. We define the time-base selection strategy for the TDoA algorithm, and we analyze the relationship between anchor deployment and positioning accuracy. The extended Kalman filter (EKF) method is presented for non-linear dynamic localization estimation, and it performs well in terms of stability and accuracy on moving targets.

## 1. Introduction

High-accuracy position information of person or device is vital for military, security, and commercial applications. For example, knowing the location information of living creatures in danger situations can help firefighters with an emergency rescue, and indoor location technology can also facilitate consumers’ shopping in the supermarket. An indoor real-time localization system (RTLS) has not yet been widely deployed, although many neoteric technologies, such as computer vision and wireless communications solutions, are adopted at [1,2]. With the increase of indoor positioning demand, how to obtain accurate location information becomes particularly important [3].

There are a few kinds of wireless sensor technologies used in indoor positioning, such as WiFi [4], RFID [5], Bluetooth Low Energy (BLE), and UWB. These technologies have their own characteristics and advantages. Among them, UWB technology has been widely used in indoor positioning in recent years. UWB (ultra wide-band) is a radio technology that uses pulse rather than the carrier to transmit data, ensuring its low-power consumption. The Federal Communications Commission (FCC) has identified that UWB pulses should occupy a broad frequency bandwidth (>500 MHz) or a relative bandwidth (>20%) with a restricted frequency band from 3.1 to 10.6 GHz and −41.3 dBm/MHz power density [6]. Ref. [7] proposes a low-complexity and noncoherent detector to detect UWB signals in the wireless sensor networks (WSNs). According to the manufacturer’s datasheets, indoor point to point measurement using UWB has high accuracy, achieving the accuracy within 10 cm. The frequency band of UWB makes the UWB devices data transmission rate up to 500 Mbit/s [8,9]. An OpenSource hardware-platform based on the DW1000 UWB chip called Wi-PoS is proposed in [10]. The PolyPoint project presents a multi-antenna plan to eliminate the influence of polarization mismatch between anchors in [11]. The authors of [12] proposes and analyzes the UWB-WBAN system, which could be applied to design for WBAN applications. In addition the authors of [13] propose an improved pilot-based algorithm based on CFO and SFO in UWB-OFDM systems with cyclic delay diversity, while [14] studies the employment of UWB in a factory and develops a Bayesian filtering solution to track the targets. The authors of [15] introduce a new low-cost RTLS without time synchronization among sensors and use a one-way communication solution to reduce the consumption of tags. Meanwhile, the authors of [16] present a unified architecture for location systems to integrate hardware, software and algorithms, while [17] proposes a semantic IIoT architecture using a communication economical RSSI/ToF ranging method.

There are several advantages in UWB technology compared to traditional wireless technologies. Due to its low equivalent isotropically radiated power emission limit, an UWB signal results in a low probability of interception and detection. Furthermore, an UWB signal has excellent multipath immunity and less susceptibility to interferences from other radios due to its wide bandwidth nature. Based on the existing UWB solutions and research mentioned above, this paper presents an UWB-based RTLS, including system architecture, wireless clock synchronization scheme, anchor deployment scheme, and time-base selection strategy. The findings made in this paper offer a solid foundation for all available UWB-based indoor localization systems design and deployment. The main contributions of this paper can be summarized as follows:

1. We design a UWB-based real-time localization system, outperforming the existing ones. According to the characteristics of UWB and TDoA positioning scheme, the system architecture of RTLS is extracted.
2. We propose a new wireless clock synchronization scheme covering both a single master anchor and multiple master anchors.
3. We present the typical deployment schemes of a single master anchor and multiple master anchors based on the principle of anchor deployment, define the time-base selection strategy for TDoA algorithm in signal master anchor and multiple master anchors systems, and reveal the relationship between anchor deployment and positioning accuracy.

To verify the performance of the real-time localization system designed in this paper and the positioning accuracy of the proposed algorithms, we have done localization experiments in a two-dimensional scenario. It is necessary to ensure that at least four anchors can receive the positioning signal sent by the tag under the line-of-sight (LoS) condition.

The rest of the paper is organized as follows. In Section 2, a literature review is given. In Section 3, a detailed description of an RTLS is given, including the architecture of real-time localization system and the UWB-based Wireless Positioning Network (U-WPN). In Section 4, we introduce the wireless clock synchronization (WCS) scheme. The localization algorithm based on TDoA and EKF will be discussed in Section 5. The reference scheme of anchor deployment and time-base selection strategy will be given in Section 6. Experiments and performance analysis are discussed in Section 7, and finally, conclusions and future work are given in Section 8.

## 2. Related Works

UWB technology has drawn enough attention to outdoor/indoor localization in recent years. Several methods are used for localization in wireless networks [18], and these approaches can generally be divided into four categories: (RSSI), (ToF), (ToA), and (TDoA). The positioning method based on TDoA has attracted more attention.

The Atlas [19] realizes a UWB-based project with DWM1000 [9]. The authors of [20] focus on TDoA-based WCS techniques, which rely on pairs of packets and a recorded timestamp, implemented in RTLS. Meanwhile, Ref. [21] proposes an effective synchronization method of the unilateral TDoA applied in ultra-wideband (UWB) localization systems. The authors of [22] describe an architecture of multi-level IoT positioning system to reduce the deployment cost, and [23] presents an E-DTDOA-based ranging algorithm used for clock drift estimation, which achieves high time resolution. In [24], the authors investigate multiple clock-drift correction methods for ToA and TDoA, in particular the DW1000 transceiver. A hybrid positioning algorithm that combines ToA and received signal strength (RSSI) measurements are presented in [25]. The authors of [26] propose a best linear unbiased estimator (BLUE) algorithm based on ultrasound TDoA measurements and investigate the geometrical dilution of precision (GDOP). An algorithm framework that integrates EKF, UKF, and PF is developed in [27]. The authors of [28] describe a UWB-based localization system using the TDoA technology. In [29], the authors propose an error-ellipse-resampling particle filter method for cooperative target tracking. A sensor network and a hybrid algorithm for tracking based on both RSS and TDoA is presented in [30]. The authors of [31] presented an algorithm to geolocate and track an unknown number of multiple emitters in the presence of clutter returns and missed detections using the TDOA technique. A derivation of the principal algorithms and an analysis of the performance of the two most important passive location systems for stationary transmitters, hyperbolic location systems and direction-finding location systems have been introduced in [32].

The positioning solutions mentioned above are algorithm oriented and do not fully consider the relationships between hardware, software, localization scheme, and anchor deployment. The RTLS proposed in this paper focuses on the shortcomings of existing solutions. The outcomes such as the architecture of a real-time localization system, the framework of a central localization engine, the wireless clock synchronization scheme, and the deployment scheme could be used as a foundation for all the available UWB-based indoor localization solutions.

## 3. System Design

This section describes the details of our new UWB real-time localization system (RTLS), including the architecture of the real-time localization system and the UWB-based Wireless Positioning Network (U-WPN).

### 3.1. Architecture of Real-Time Localization System

**Figure 1:**
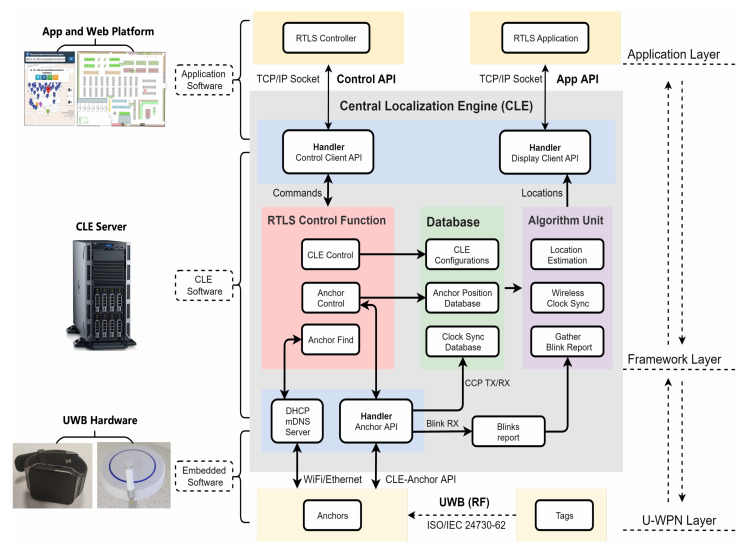
Architecture of Real-Time Localization System.

Figure 1 gives a detailed view of the architecture of the real-time localization system. The UWB-based RTLS can be regarded as a three-layer architecture, including application layer, CLE (central localization engine) framework layer, and the U-WPN layer. UWB anchors and tags are working at the U-WPN layer, and the UWB messages could be transmitted between them through ISO/IEC defined protocol. The CLE has a control function unit, a database, and an algorithm unit, which realizes the function of wireless clock synchronization and location estimation. Mobile apps and web platforms could interact with the CLE framework layer through the open APIs.

### 3.2. UWB-Based Wireless Positioning Network

**Figure 2:**
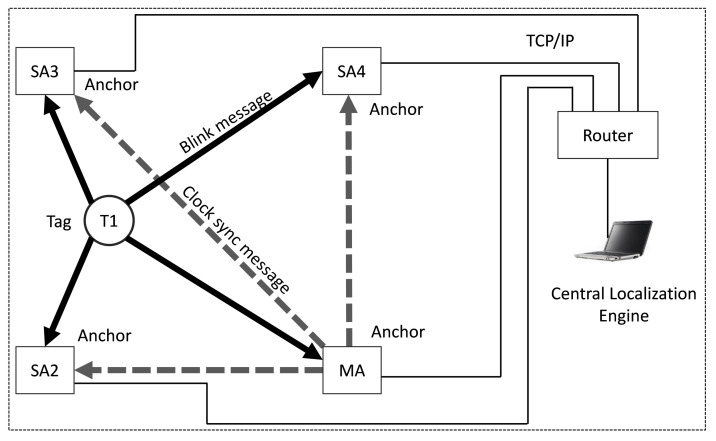
UWB-based wireless positioning network.

Figure 2 demonstrates the UWB-based Wireless Positioning Network (**U-WPN**), which exchanges the UWB messages and Ethernet communication for a system with four anchors, one tag, and one **CLE** (central localization engine). MA is the master anchor, and SA2, SA3, SA4 are the slave anchors. T1 is the tag, and the **CLE** runs on an upper computer. The tag transmits a periodic blink frame, which is received and timestamped at the anchors. Each anchor then sends the ToA reports to a central localization engine (**CLE**), and the **CLE** uses the ToAs to estimate the tag’s location. Tags periodically send “blink” messages, which are received by all anchors in the range.

To ensure that the ToAs recorded by the anchors are on the same reference clock, we need to eliminate the clock offset and drift of anchors. It is called clock synchronization and is typically achieved via wired clock distribution to the anchors. As an alternative to using a wired timer, the TDoA-based RTLS designed in this paper includes a wireless clock synchronization algorithm that employs UWB messages sent between anchors to correct clock drift and offset.

Anchors can be configured as master anchors (MA) and slave anchors (SA). MA transmit Clock Calibration Packets (CCP) periodically. Slave anchors receive these CCP and report their reception to the **CLE** to track the relative clock offset between the sending master anchors and the receiving slave anchors. If an RTLS has more than one master, it could be called a multiple masters-based RTLS. A “secondary” master can delay sending its CCP by a configured lag time after the reception of a CCP from a “primary” master anchor to prevent from CCP collisions between CCP transmissions.

The purpose of clock synchronization is to record each anchor’s clock and calibrate the blink message timestamps to a recorded timestamp. The **CLE** also performs the TDoA algorithm to estimate the tags’ locations.

## 4. Wireless Clock Synchronization

Each UWB device is equipped with a high-resolution timer. The oscillation frequency will drift over time, and we must eliminate the effects of drift by clock calibration. The clock frequency offset and instability significantly impact the positioning accuracy in an RTLS. Most of the RTLS utilize a time measurement to get ToA or TDoA, which is used for position calculation. All the anchors need to be synchronized, as the precise timestamp is essential for location estimation. Three main issues should be addressed in synchronization [33]:

- **Offset synchronization**—Ensure the recorded timestamp between anchors using the same reference time;
- **Drift compensation**—Eliminate frequency deviation caused by temperature and other environmental factors;
- **Antenna delay calibration**—Eliminate the changes of internal propagation delay of UWB devices.

The measurement delay in the timestamp includes transmitting antenna delay and receiving antenna delay. These antenna delays are specifically internal to the chip and have not been included in time of flight (ToF). The solutions proposed in [20,34] can be used for antenna delay calibration.

The wired clock synchronization scheme is a universal solution. However, additional clock synchronizing timer and transmission lines add to the difficulty of anchor deployment, so it is not suitable for complex environments. Hence, a wireless clock synchronization solution without extra equipment is urgently needed. The method proposed in [20] relies on the pair of packets and a known recorded timestamp, which uses the remained nodes’ corrected timestamps to carry out WCS. Meanwhile, a simple clock model is used in [19] for wireless clock correction based on the offset and the drift. It is necessary to retain clock models for each anchor. The authors of [35] propose a novel wireless clock synchronization scheme that can be used in multi-user systems to overcome the limitations of TWR-based positioning.

There are two types of synchronization protocols: one-way synchronization and two-way synchronization. In the TDoA-based positioning scenario, a one-way time transfer protocol is adopted in this paper. The algorithm of linear interpolation **(LI)** [36] is simple and effective, but it is not suitable for real-time positioning scenarios. The algorithms of proportional–integral–differential **(PID)** [37] control and Kalman filter **(KF)** [38] can be used to predict the ToA between the tag and the anchor. The wireless clock synchronization scheme adopted in this paper is a combination of a linear interpolation algorithm and Kalman filter algorithm **(LI-KF)**. Firstly, the raw ToA is corrected by linearly interpolating between the ToAs of the synchronization messages. Then, the Kalman filter is used to retrieve and update the latency between synchronization periods.

Clock offset correction is easier to solve when we know each anchor’s reference clock’s deviation, but the clock drift is not easy to eliminate due to the different clock modules in the anchors. Figure 2 depicts a basic UWB-based Wireless Positioning Network **U-WPN** in Section 3. We will use the **U-WPN** to introduce our proposed wireless clock synchronization scheme in this section. Each anchor and tag has its own timer, and they are un-synchronized. The exact clock information of another device is unknown, which can only be obtained through a timestamp. The precise distance between the sending device and the receiving device is unknown. If all the slave anchors could receive the **CCPs** sent by one master anchor, then we adopt the scheme of **WCS** with a single master. If the **CCP** sent by a master anchor cannot be received by all slave anchors, multiple master anchors need to be deployed in the location area. In that case, the **WCS** with multiple masters needs to be adopted for completed coverage.

### 4.1. Wcs with a Single Master

**Figure 3:**
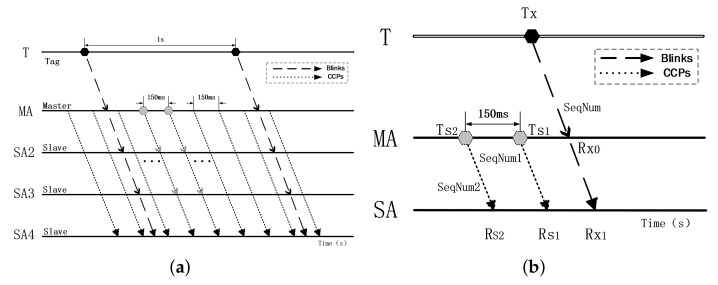
Diagram of WCS: (**a**) The overall diagram of WCS with a single master, (**b**) the diagram of the details of WCS.

The overall diagram of WCS with a single master is demonstrated in Figure 3a, which shows our proposed positioning system receiving and sending positioning and synchronization messages on the timeline. **T** is tag, **MA** is a master anchor and **SA2–SA4** are slave anchors. There are five timelines, with the top one representing the tag and the bottom four belonging to the individual anchors. The dark dashed lines represent the positioning packet (**Blinks**) sent by the tag with a sending period of 1 s, while the light dashed lines represent the synchronous packet (**CCPs**) sent by the master anchor at an interval of 150 ms.

Once the tag sends the **Blink** or the master anchor sends the **CCP**, the slave anchors in the corresponding deployment area will receive the **Blink** or **CCP** and record the timestamp in the anchor’s clock system, respectively. For the master anchor, in addition to receiving the Blink from the tag similar to a slave anchor, it is also necessary to periodically send the **CCP** and record the sending timestamp.

To illustrate the process of clock synchronization in detail, we use a simple case with one tag (T), one master anchor (MA), and one slave anchor (SA), as shown in Figure 3b. After receiving the **Blink**, **MA** and **SA** will record the received timestamp ($R_{x0}$, $R_{x1}$) and serial number (**SeqNum**) of this **Blink**. Meanwhile, the **MA** will record the sending timestamp ($T_{s1}$, $T_{s2}$) and the corresponding serial number (SeqNum1, SeqNum2) when it sends the **CCP**. When the **CCP** reaches the **SA** node, the timestamp ($R_{s1}$, $R_{s2}$) and the corresponding serial number will also be recorded.

**Figure 4:**
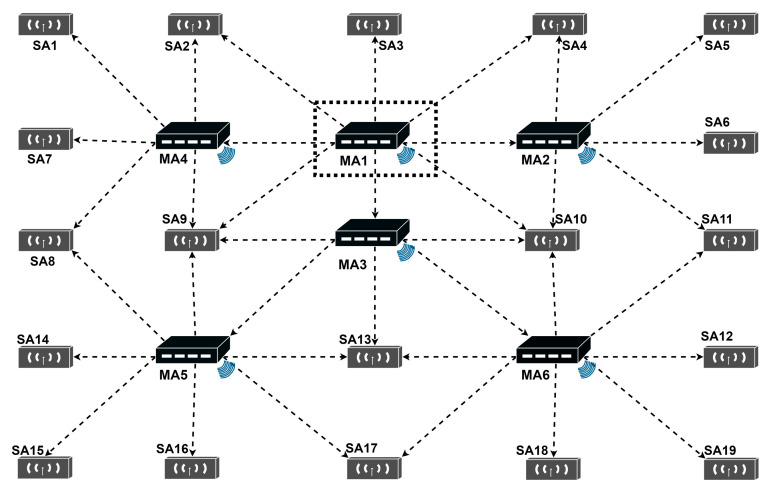
Diagram of WCS with multiple masters.

The system clock drift caused by quartz crystal illustrates a certain regularity, so we build a scale coefficient model to correct the clock drift. As shown in Figure 4, an original TDoA can be expressed as: $TDoA_{raw}=R_{x1}-R_{x0}$, and the scale coefficient of calibration can be set as *K*.

(1) $$K=\frac{T_{s1}-T_{s2}}{R_{s1}-R_{s2}}$$

The corrected TDoA is $TDoA_{sync}$:

(2) $$TDoA_{sync}=K*TDoA_{raw}$$

where $R_{x1}$ and $R_{x0}$ are the timestamps recorded by **SA** and **MA** when they receive a **Blink**, $R_{s1}$ and $R_{s2}$ are the timestamps recorded by **SA** when it receives the **CCPs**, $T_{s1}$ and $T_{s2}$ are the timestamps recorded by **MA** when it sends the **CCPs**. Finally, the Kalman filter will be adopted to trace each anchor’s clock offset after gathering all the **$TDoA_{sync}s$**. Based on this scheme, the clock of each anchor can be synchronized.

### 4.2. WCS with Multiple Masters

The overall diagram of WCS with multiple masters is demonstrated in Figure 4, including one primary **MA** (**MA1**), five secondary **MAs** (**MA2–MA6**), and nineteen **SAs**. The **CCPs** between anchors (**MA** and **SA**, or **MA** and **MA**) are in dotted lines. The deployment of **MAs** has been made to ensure that all secondary master anchors can communicate with the master anchor of the upper level, and any slave anchor can receive the synchronization signal of at least one master anchor. The remainder of this section describes in detail how the WCS scheme with multiple masters works.

In order to cover a large area, it is necessary to employ more than one **MA**, where each **MA** is used as a reference to correct the clock drift of its neighboring **SAs**. We establish a multi-level cascade topological structure of the **MAs** to coordinate the order of sending **CCPs** between the primary **MAs** and the secondary **MAs** in a complete clock synchronization.

**MA1** is the primary master anchor. Any **MA** receiving the **CCPs** sent by **MA1** is the secondary master anchor. In addition, the **MA** receiving the **CCPs** of the secondary master anchor is the level-3 master anchor. So, **MA2**, **MA3** and **MA4** are secondary master anchors, and **MA5** and **MA6** are level-3 master anchors. According to this rule, the cascading model of MA can be obtained as follows: primary master anchor −> secondary master anchor −> level-3 master anchor −> level-4 master anchor −>…−> level-N master anchor. When a lower-level master anchor receives the **CCP** from its upper level master anchor, it starts to send the **CCP** after a short interval (Lag).

**Figure 5:**
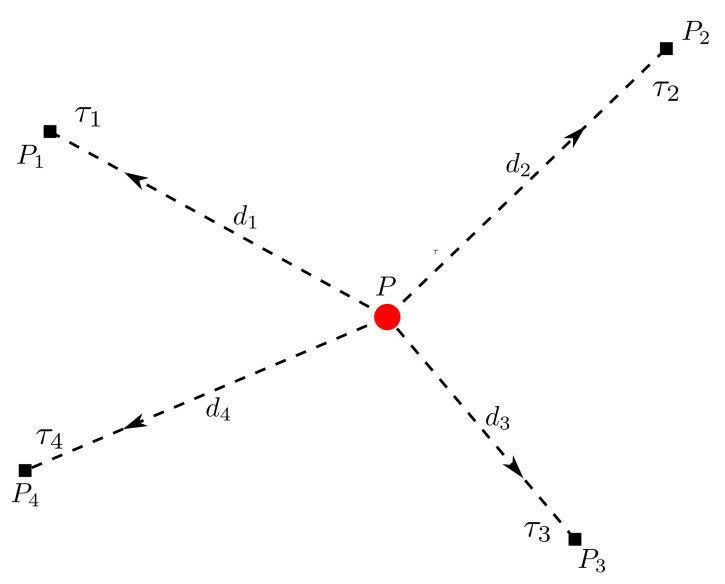
The model of TDoA.

Figure 5 is an example to illustrate WCS with multiple master anchors. Surrounding the primary master anchor (**MA1**) are three secondary master anchors (**MA2**, **MA3**, and **MA4**) and five slave anchors **(SA2**, **SA3**, **SA4**, **SA9**, and **SA10**), and they are configured to follow **MA1**, so their clocks need to be synchronized with **MA1**. There are five slave anchors (**SA1**, **SA2**, **SA7**, **SA8**, and **SA9**) around the secondary master anchor **MA4**, and the slave anchors are configured to follow **MA4**, so their clocks could be synchronized with **MA4**. The slave anchors could be configured to follow more than one master anchors, such as **SA2**, **SA3**, **SA4**, **SA8**, **SA9**, and **SA10**.

Based on the topology of the multi-level master anchor scheme, we summarize the strategies of WCS with multiple masters:

- Setting every master anchor to send **CCPs** in a specified interval;
- Choosing one master anchor as the primary master anchor (there is one and only one primary master anchor in an RTLS);
- Setting the rest of master anchors as different levels;
- Setting the lower-level master anchors to follow its upper-level master anchor;
- Setting the lower-level master anchors to send **CCPs** with different lags to avoid the collision (for example, **MA2** delay one lag, **MA3** delay two lags, and **MA4** delay three lags);
- Setting the slave anchors to follow the master anchor (ensure the slave anchors could receive the **CCPs** sent by its master anchor);
- Collecting the recorded ToAs of all the anchors and the transmiting timestamps of master anchors;
- Using the WCS method shown in Section 4.1 to synchronize the TDoAs.

## 5. Location Estimation Based on TDoA

### 5.1. Model

TDoA-based localization is a common approach used in the UWB system. The positioning system includes several anchors and tags. Assume there is only a single tag to be localized. The tag transmits signals to the anchors periodically. The model of TDoA can be expressed in Figure 5.

The tag could transmit the positioning frame to the anchors and propagate in a straight line to the anchors (line of sight condition should be satisfied, LoS). For two-dimension positioning, assuming that $p={\left(x,y\right)}^{T}\in R^{2}$ is the coordinates of the target, where $p_{i}={\left(x_{i},y_{i}\right)}^{T}\in R^{2},i=1,2,\ldots ,n$ are the coordinates of anchors, and $d_{i}=||p-p_{i}\left|\right|$ are the distances between the tag and anchors.

We could get the timestamp $\tau_{i}$ when the frame is received by the anchors, assuming that the measurement of ${\hat{\tau }}_{i}$ satisfies $\hat{\tau_{i}}∼N\left(\tau_{i},\delta_{i}^{2}\right)$. The core formula of TDoA is

(3) $${\hat{d}}_{ij}\overset{\Delta }{=}{\hat{d}}_{i}-{\hat{d}}_{j}=c\left({\hat{\tau }}_{i}-{\hat{\tau }}_{j}\right):=c{\hat{\tau }}_{ij},\;\forall \;i,j=1,2,\ldots ,n$$

where (*c*) is the speed of light, the range differences (RD) represent the TDoA measurements, and the distance ${\hat{d}}_{ij}$ of here satisfy $\sum_{i,j}{\hat{d}}_{ij}\equiv 0$.

The aim is to find *p*, so

(4) $$d_{ij}\left(p\right)={\hat{d}}_{ij},\;\forall \;i,j=1,2,\ldots ,n$$

where $d_{ij}\left(p\right)=d_{i}\left(p\right)-d_{j}\left(p\right)=\left|\right|p-p_{i}\left|\right|-\left|\right|p-p_{j}\left|\right|$.

The inputs of the method are the anchors’ coordinates, $p_{i}$, the measured TDoA, ${\hat{\tau }}_{ij}$, and the outputs are the coordinates of the tag, *p*.

Because of the measurement error, the least square condition is usually considered to estimate the position $\hat{p}$.

(5) $$\hat{p}=\underset{\begin{array}{c} p \end{array}}{\mathrm{argmin}}{\sum_{i,j=1,2,\ldots ,n}\left|\right|d_{ij}-{\hat{d}}_{ij}\left|\right|}^{2}.$$

### 5.2. The Algorithm of EKF

Location estimation through EKF is available in [25], and a constant velocity (CV) model is selected to describe the RTLS designed in this paper. The state equations and update equations of the EKF model are illustrated in the following formulas. The inputs of EKF include the range difference (RD) $\hat{d_{ij}}$, the coordinates of anchor $p_{i}$, and the initial position of the tag *p*. Meanwhile, the state transfer matrices and other covariance matrices used in the EKF algorithm could be calculated through the References [19,25].

1. **The Process Model**
(6) $$X_{k}=f\left(X_{k-1}+u_{k-1}\right)+W_{k}$$
(7) $$Z_{k}=h\left(X_{k}\right)+V_{k}$$
2. **Time Update (“Predict”)**
(8) $${\hat{X}}_{k}^{˘}=F{\hat{X}}_{k-1}+B_{k}u_{k-1}$$
(9) $$P_{k}^{˘}=FP_{k-1}F^{T}+Q$$
3. **Measurement Update (“Correct”)**
(10) $$K_{k}=P_{k}^{˘}H_{k}^{T}{\left(H_{k}P_{k}^{˘}H_{k}^{T}+R\right)}^{-1}$$
(11) $${\hat{X}}_{k}={\hat{X}}_{k}^{˘}+K_{k}\left(Z_{k}-h\left({\hat{X}}_{k}^{˘}\right)\right)$$
(12) $$P_{k}=\left(I-K_{k}H_{k}\right)P_{k}^{˘}$$

where

- $X_{k}$ is the true state vector, $X_{k}={\left[x,y,v_{x},v_{y}\right]}^{T}$, where *x* and *y* represent the coordinates of the tag’s position *p*, $v_{x}$ and $v_{y}$ represent the velocities along the *x* and *y* directions, the non-linear state function *f* is used to determine the predicted state from the previous state.
- $h\left(X_{k}\right)$ is a function of the state vector, which can be defined as Formula (13),
  (13) $$h\left(X_{k}\right)={\left[\begin{array}{c} d_{21}\\ d_{31}\\ \ldots \\ d_{n1} \end{array}\right]}_{(n-1)\times 1}={\left[\begin{array}{c} d_{2}-d_{1}\\ d_{3}-d1\\ \ldots \\ d_{n}-d_{1} \end{array}\right]}_{(n-1)\times 1}$$
  where $d_{n}$ is the distance between the anchor *n* and the tag *p*, $d_{1}$ is the distance between the reference anchor $p_{1}$ (as shown in Figure 5, we could regard $p_{1}$ as the reference anchor) and the tag *p*, and $d_{n1}$ denotes the range difference of $d_{n}$ and $d_{1}$.
- $Z_{k}$ is the observation vector, which only depends on the measurements of sensors (the range differences between the tag and anchors), so $Z_{k}={\left[d_{21}\;d_{31}\;\ldots \;d_{n1}\right]}^{T}$.
- ${\hat{X}}^{˘}$ is a prior estimated state vector; ${\hat{X}}_{k}$ expresses a posteriori estimate, which is the linear combination of the prior estimate ${\hat{X}}^{˘}$, $Z_{k}$, and $h({\hat{X}}^{˘})$.
- Δt denotes the time elapsed between the previous estimation time and the current time, and Δt=0.1 s, considering the relationship between position and velocity in the 2D scene based on the CV model, because
  (14) $$\left[\begin{array}{c} x_{k}\\ y_{k}\\ v_{x_{k}}\\ v_{y_{k}} \end{array}\right]=F\left[\begin{array}{c} x_{k-1}\\ y_{k-1}\\ v_{x_{k-1}}\\ v_{y_{k-1}} \end{array}\right]=\left[\begin{array}{c} x_{k-1}+v_{x_{k-1}}\Delta t\\ y_{k-1}+v_{y_{k-1}}\Delta t\\ v_{x_{k-1}}\\ v_{y_{k-1}} \end{array}\right]$$
  so the state transition matrix *F* can be expressed as:
  (15) $$F=\left[\begin{array}{cccc} 1 & 0 & \Delta t & 0\\ 0 & 1 & 0 & \Delta t\\ 0 & 0 & 1 & 0\\ 0 & 0 & 0 & 1 \end{array}\right]$$
- $B_{k}$ is the input control matrix and the size is 4×2, and $u_{k}$ is the input control vector and the size is 2×1. For a simple system without external control, this part can be ignored, so the item $B_{k}u_{k}$ is zero.
- The $P_{k}$ is the estimated covariance matrix, which can be recursively derived from the initial matrix $P_{0}$. The covariance matrix $P_{0}$ is related to the initial state vector. $P_{k}^{˘}$ is a prior estimate $P_{k}$, and $K_{k}$ is the Kalman gain.
  (16) $$P_{0}=\left[\begin{array}{cccc} \sigma_{x_{0}}^{2} & 0 & 0 & 0\\ 0 & \sigma_{y_{0}}^{2} & 0 & 0\\ 0 & 0 & \sigma_{v_{x0}}^{2} & 0\\ 0 & 0 & 0 & \sigma_{v_{y0}}^{2} \end{array}\right]$$
  where $\sigma_{x_{0}}^{2}$, $\sigma_{y_{0}}^{2}$, $\sigma_{v_{x0}}^{2}$, and $\sigma_{v_{x0}}^{2}$ represent the initial variances of the state vector components.
- The CV model assumes that the tag moves at a constant velocity. In the problem of tag state prediction, it is obvious that the tag does not necessarily move at a constant velocity. Therefore, our process model includes the uncertainty of tag position and describes this uncertainty in the *Q* matrix. In the kinematic equation, we express this uncertainty in terms of acceleration *a*, $a=\frac{\Delta v}{\Delta t}=\frac{v_{k}-v_{k-1}}{\Delta t}$, the velocity is not uniform, and acceleration is not known, so it can be added to process noise.
  (17) $$Gu=\left[\begin{array}{cc} \frac{\Delta t^{2}}{2} & 0\\ 0 & \frac{\Delta t^{2}}{2}\\ \Delta t & 0\\ 0 & \Delta t \end{array}\right]\left[\begin{array}{c} a_{x}\\ a_{y} \end{array}\right]=\left[\begin{array}{c} \frac{a_{x}\Delta t^{2}}{2}\\ \frac{a_{y}\Delta t^{2}}{2}\\ a_{x}\Delta t\\ a_{y}\Delta t \end{array}\right]$$
  where $a_{x}$ and $a_{y}$ indicate the acceleration in the x and y directions, respectively, *G* is the input control matrix without a random variable and the size is 4×2, and *u* is the input control vector with random acceleration and the size is 2×1. So, the covariance matrix of process noise $Q=GG^{T}\sigma^{2}$, and the size of *Q* is 4×4, $\sigma^{2}$ is set to 0.5 m/s${}^{2}$ according to the movement of the tag. Because the process noise is randomly substituted, $W_{k}$ follows Gaussian distribution: $W_{k}∽N\left(0,Q\right)$.
- In the real-time localization system based on UWB, each set of measurements is affected by random noise. *R* represents the covariance matrix of observation noise $R=diag(\omega_{1}^{2},\;\omega_{2}^{2}\;\ldots \;\omega_{n-1}^{2})$, and the size is (n−1)×(n−1), the parameters ($\omega^{2}$) of the random noise measurement matrix can be provided by the sensor manufacturer, and $V_{k}$ follows $V_{k}∽N\left(0,R\right)$.
- $H_{k}$ represents the Jacobian matrix related to expected measurements, which can be expressed as:
  (18) $$H_{k}={\left[\begin{array}{cccc} \frac{\partial h_{2}\left(\hat{X_{k}}\right)}{\partial x} & \frac{\partial h_{2}\left(\hat{X_{k}}\right)}{\partial y} & 0 & 0\\ \ldots & \ldots & \ldots & \ldots \\ \frac{\partial h_{n}\left(\hat{X_{k}}\right)}{\partial x} & \frac{\partial h_{n}\left(\hat{X_{k}}\right)}{\partial y} & 0 & 0 \end{array}\right]}_{(n-1)\times 4}$$
  The element of the Jacobian matrix $H_{k}$ can be described as:
  (19) $$\frac{\partial h_{n}\left(\hat{X_{k}}\right)}{\partial x}=\frac{\hat{x}-x_{n}}{\hat{d_{n}}}-\frac{\hat{x}-x_{1}}{\hat{d_{1}}}$$
  (20) $$\frac{\partial h_{n}\left(\hat{X_{k}}\right)}{\partial y}=\frac{\hat{y}-y_{n}}{\hat{d_{n}}}-\frac{\hat{y}-y_{1}}{\hat{d_{1}}}$$
  where $\hat{d_{n}}=ED\left(\hat{p},p_{n}\right)$ is the estimated Euclidean distance between the tag and the n-th UWB anchor, and $\hat{p}$ is the estimate position.
  (21) $$ED\left(\hat{p},p_{n}\right)=\sqrt{{\left(x_{n}-\hat{x}\right)}^{2}+{\left(y_{n}-\hat{y}\right)}^{2}}.$$

## 6. Time-Base Selection Strategy and Anchor Deployment Scheme

### 6.1. Time-Base Selection Strategy

**Figure 6:**
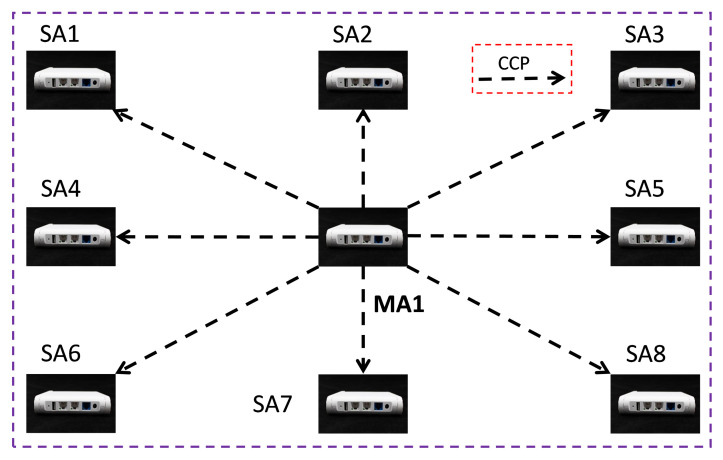
Diagram of anchor deployment with a single master.

We need a time-base selection strategy to cover different anchor deployment schemes in the TDoA-based real-time localization system, where time-base means the reference anchor’s ToA which will be selected to obtain the TDoA ($TDoA=ToA_{i}-ToA_{reference}$). The anchor deployment based on WCS with a single master is illustrated in Figure 6; we choose the recorded timestamp of the single master anchor, **MA1**, as the time-base of TDoA.

We define the time-base selection rules with multiple master anchors as follows:

- If the **CCP** received by a **SA** came from the same **MA**, the recorded timestamp of the **MA** is selected as the time-base of TDoA.
- If the **CCP** received by a **SA** came from two or more **MAs**, then the recorded timestamp of the **SA** is selected as the time-base of TDoA.

**Figure 7:**
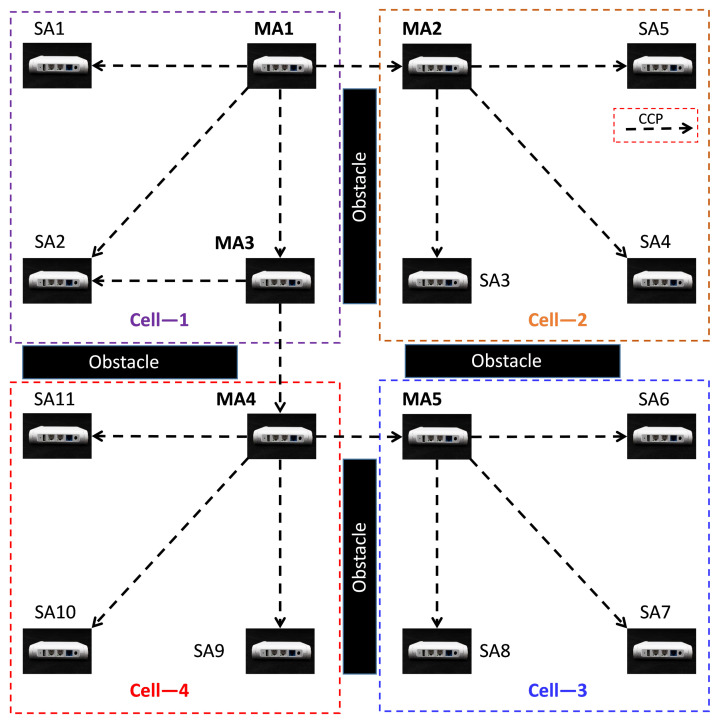
Diagram of anchor deployment with multiple masters.

As an example, the anchor deployment based on WCS with multiple masters is demonstrated in Figure 7; when the tag is located in Cell-1, **SA2** could receive the **CCPs** from both **MA1** and **MA3**, so the recorded timestamp of **SA2** is selected as the time-base of TDoA; when the tag moves to Cell-2, the **SAs** in this region can only receive **CCPs** from **MA2**, so the recorded timestamp of **MA2** is selected as the time-base of TDoA.

### 6.2. Anchor Deployment Scheme

In a real deployment scenario, the necessary deployment rules need to be followed to ensure positioning accuracy. Corresponding to the two WCS schemes proposed in Section 4, we present two typical reference deployment schemes in practical scenarios.

The anchor deployment based on WCS with a single master is illustrated in Figure 6. We consider the case of a single master anchor covering a larger area, so the number of anchors go up to eight. To ensure smooth communication between the master anchor and all the surrounding slave anchors, we put the master anchor at the center of the positioning area.

In Figure 7, we discuss the situation of the location area with multiple master anchors. To make it easy to describe, we assume that the tracking space is divided into four separate areas, including one primary master anchor MA1, four secondary master anchors, MA2–MA5, eleven slave anchors, SA1–SA11. The dotted line indicates the CCP, representing the synchronous message. In the case of the current reference anchor deployment, the WCS with multiple masters scheme is adopted.

### 6.3. Common Deployment Rules

The following seven rules extracted from our experiments should be considered when deploying the anchors.

1. Keep a LoS (line of sight) between a master anchor and slave anchors (at least three slave anchors).
2. The anchors should be installed above the localized objects. Assure a clear line of sight between tags and anchors. Do not hide the tag behind materials that attenuate the radio signal such as water, a human body, or metal parts.
3. Mount anchors (surrounding master anchor) ideally at the same height (keep a variation of 1 m maximum).
4. Keep anchors detached away from walls or ceilings (ideally 50 cm but not less than 15 cm—shorter detachment may cause higher signal attenuation and inaccuracies due to reflections).
5. Keep a square geometry when designing anchor deployment. The minimum distance between anchors should be longer than 3 m. The location area should be bigger than 3 m × 3 m.
6. Orient the anchors such that their radiation capabilities are satisfactory.

### 6.4. Dilution of Precision (DoP) Guided Deployment

The DoP model could be used for evaluating the relationship between the anchors’ placement geometry with the positioning accuracy of the RTLS. The DoP model could be adapted to measure various positioning systems’ performance and is independent of communication technologies and modes. We have studied the DoP for the TDoA technique with respect to anchor deployment in [39].

The DoP provides a gain factor that is numerically dimensionless and represents the relationship between the measurement error at a given position and the geometry of the anchors. It should be noted that the relationship between anchor spacing and DoP is fragile, but their geometric structure will have a specific influence. So, it is worth discussing how to deploy anchors based on the DoP. We can adopt the most practical horizontal DoP (HDoP) because 2D positioning is used much more frequently than 3D positioning in most scenarios. This work mainly considers square anchor geometry.

## 7. Experiments and Performance Analysis

### 7.1. Introduction of the Experimental Environment

**Figure 8:**
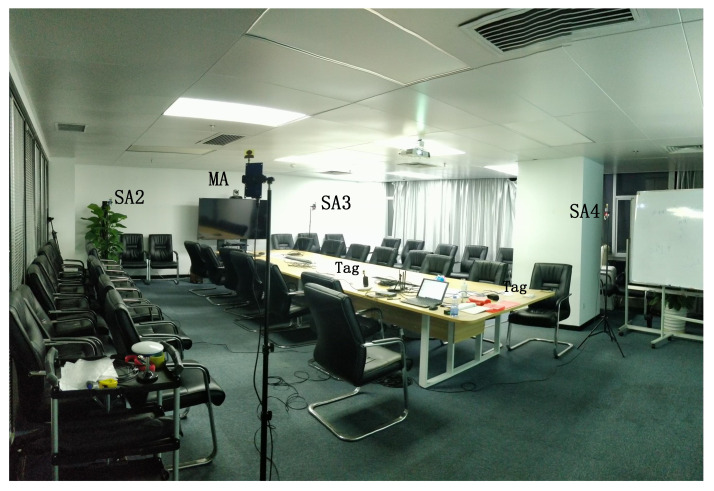
System implementation.

According to the UWB-based Wireless Positioning Network (U-WPN) proposed in Section 3, a test experiment is set up to analyze the proposed approach’s positioning accuracy and stability, which is shown in Figure 8. We deploy four anchors in a conference room. They are fixed on the four vertices of a rectangle with 6 m length and 4 m width. The RTLS we have built includes four anchors, two tags, one router, one PoE switch, and a **CLE**. The test process is as follows: four anchors are fixed on the tripods with the same height of 1.80 m. The sending periods of **CCP** and **Blink** are set, respectively.

- A CCP is transmitted from the master anchor every 150 milliseconds.
- A blink is transmitted from the tag every 100 milliseconds.

For the static location, we use the root mean square error (RMSE) of the Euclidean distance between the actual position and the estimated position. That is

(22) $$RMSE=\sqrt{\frac{1}{N}\sum_{k=0}^{N}{\left(x_{k}-x\right)}^{2}+{\left(y_{k}-y\right)}^{2}}$$

where *N* is the number of sample points $\left(x_{k},y_{k}\right)$ is the estimated position $p_{k}$ and (x,y) is the actual target location.

For the tracking problem, we calculate the RMSE between the estimated location and the distance to the reference moving line, namely,

(23) $$RMSE=\sqrt{\frac{1}{N}\sum_{k=0}^{N}{d(p_{k},L)}^{2}}$$

where *N* is the number of tracking points, and $d(p_{k},L)$ is the Euclidean distance between the estimated point $p_{k}$ and the reference path *L*.

### 7.2. Performance of WCS Scheme with a Single Master Anchor

**Figure 9:**
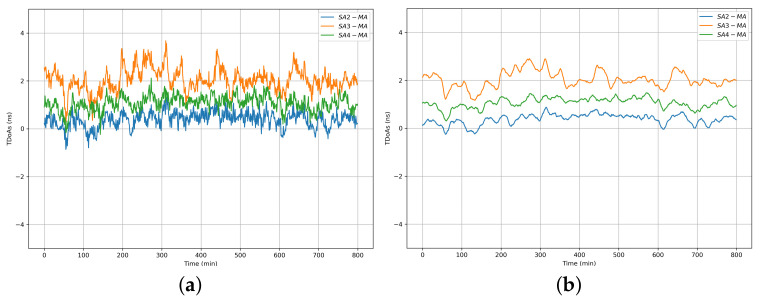
Performance of WCS: (**a**) Performance of WCS with a signal master anchor, (**b**) Performance of WCS with a signal master anchor based on KF.

The results of the WCS scheme with a single master anchor are shown in Figure 9a,b. **SA2-MA**, **SA3-MA**, and **SA4-MA** represent the TDoA between the master anchor and the three slave anchors, respectively. It is obvious that the original synchronized TDoA data demonstrated in Figure 9a have large fluctuations and synchronization errors of several nanoseconds. Such TDoA data cannot be directly used for precise positioning. We used the Kalman filter (KF) to process the original synchronized TDoA data, and the results are illustrated in Figure 9b. It is obvious that KF greatly reduces the influence of noise on the WCS scheme.

For each **SA**, the Blink RX timestamps are corrected with the wireless clock synchronization algorithm. The corrected timestamps, which follow its corresponding master recorded timestamp, are used for calculating the TDoA of each Blink message between the **MA** and each **SA**. The standard deviations of the TDOA for the deployed three slave anchors in the test environment were 0.18 ns, 0.19 ns, and 0.14 ns throughout an 800-min test. The average value of these deviations is not more than 200 ps, representing a standard deviation of position less than 6 cm.

### 7.3. Performance of WCS Scheme with Multiple Master Anchors

**Figure 10:**
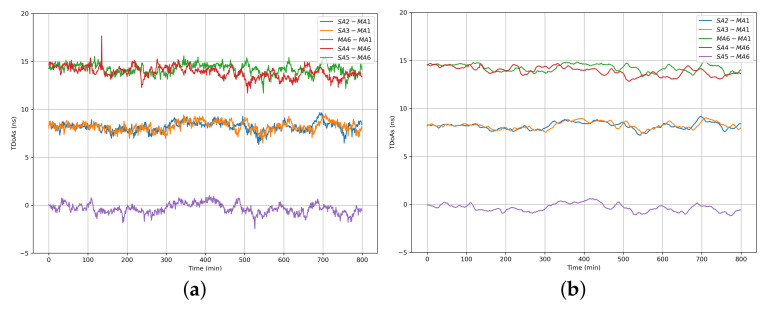
Performance of WCS: (**a**) Performance of WCS with multiple master anchors, (**b**) Performance of WCS with multiple master anchors based on KF.

The results of the WCS scheme with multiple master anchors are shown in Figure 10a,b. **SA2-MA1**, **SA3-MA1**, **MA6-MA1**, **SA4-MA6**, and **SA5-MA6** represent the TDoAs between the master anchors and slave anchors, respectively. The original synchronized TDoA data are demonstrated in Figure 10a, and the results after applied KF are illustrated in Figure 10b.

**Figure 11:**
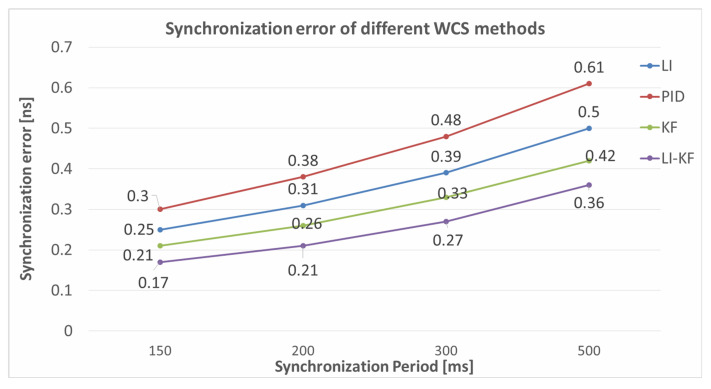
Comparative diagrams of different WCS methods.

The wireless clock synchronization algorithms using linear interpolation (LI) [36], proportional-integral-differential (PID) [37], Kalman filter (KF) [38], and linear interpolation based Kalman filter (LI-KF) have been compared in this paper. The results are demonstrated in Table 1 and Figure 11.

### 7.4. Performance of RTLS with a Single Master Anchor

**Figure 12:**
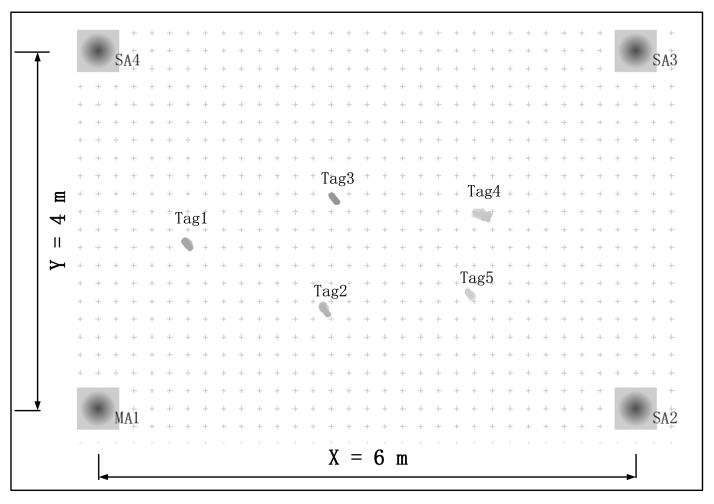
Result of RTLS with a single master anchor.

**Figure 13:**
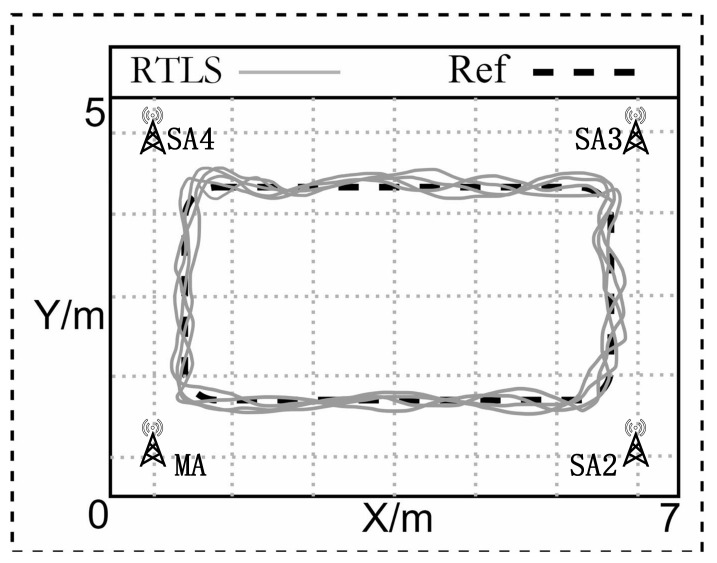
Result of tag tracking with a single master anchor.

We use an Extended Kalman Filter (EKF) in the RTLS to estimate the tags’ positions, and the test results are demonstrated in Figure 12. The test results of tag tracking with a single master anchor are illustrated in Figure 13. There are totally 4 anchors in the current deployment scenario, the location of the each anchor is shown in Table 2.

### 7.5. Performance of RTLS with Multiple Master Anchors

**Figure 14:**
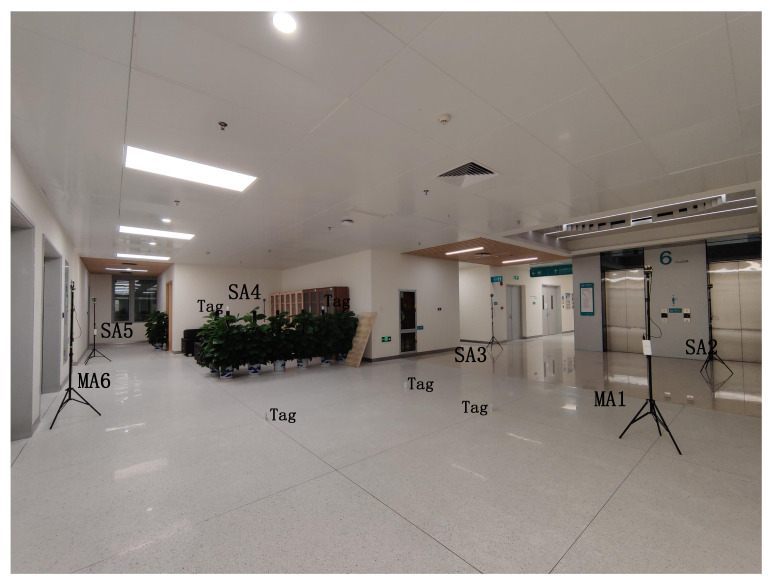
System implementation with multiple master anchors.

Another test experiment is set up to analyze the proposed approach’s positioning accuracy and stability, which is shown in Figure 14. The test is conducted in a 9.88-m wide and 11.02-m long Hall. We deploy two master anchors (**MA1**, and **MA6**) and four slave anchors (**SA2**, **SA3**, **SA4**, and **SA5**). In addition, there are five tags (**Tag1**, **Tag2**, …, **Tag5**) deployed in this area. There are six anchors in this scenario; the location of each anchor is shown in Table 3.

**Figure 15:**
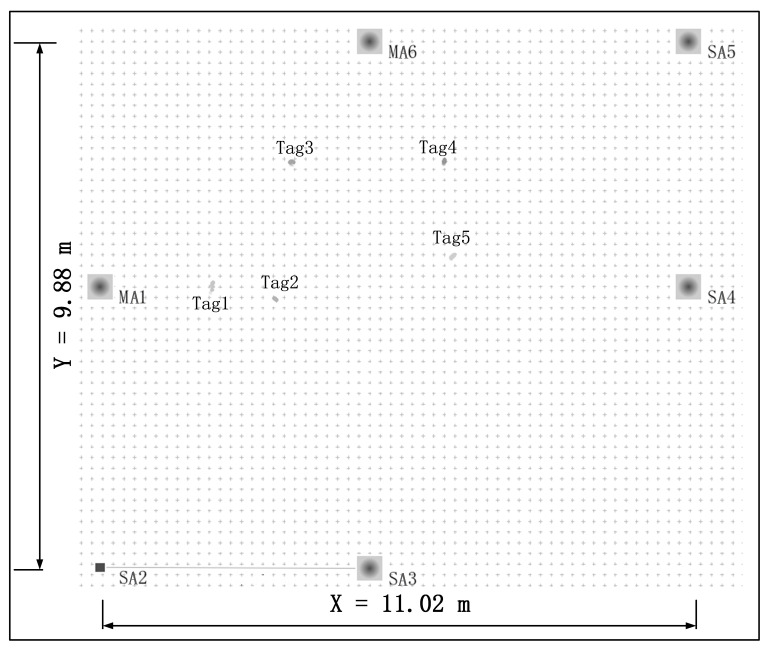
Result of RTLS with multiple master anchors.

**Figure 16:**
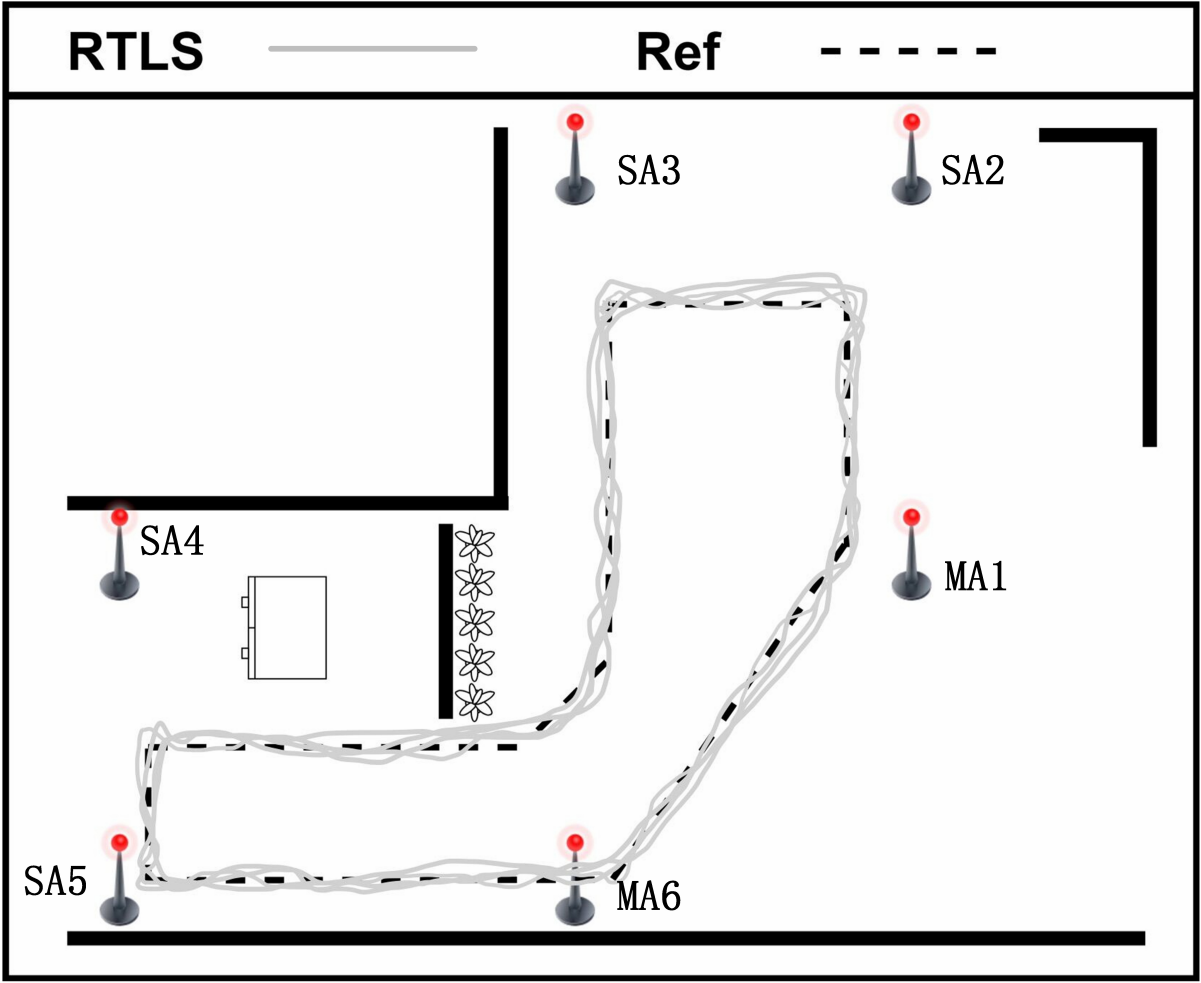
Result of tag tracking with multiple master anchors.

**Figure 17:**
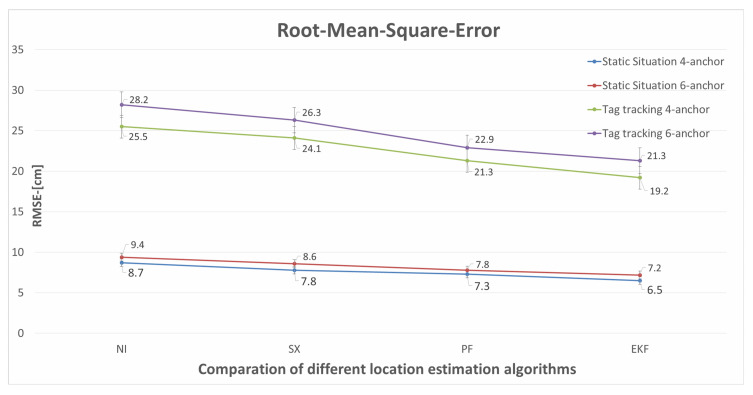
Diagram of different location estimation algorithms.

We use the RTLS built in this paper to estimate the tags’ positions in a static scenario, and the test results are demonstrated in Figure 15. The test results of tag tracking with multiple master anchors are demonstrated in Figure 16. The Newton iteration **(NI)** method [40] is used to solve non-linear equations, and linear solutions are used to constrain non-linear solutions after selecting appropriate initial value points. The spherical-intersection **(SX)** [41] algorithm does not require any prior information and has low complexity, which is a closed-form solution for source location that is presented given time-of-arrival difference measurements when the distance from the source to any arbitrary reference is unknown. Particle filter **(PF)** [27] proposed a probabilistic method on how to evaluate the robot state in a prior defined map, which solves the problem of localization as a Bayesian filtering problem to guesstimate the posterior or subsequent density of the state consuming weighted particles. The location estimation algorithms using Newton iteration, spherical-intersection, particle filter, and extended Kalman filter **(EKF)** have been compared in this paper; the results of RMSE for static situation and tag tracking are illustrated in Table 4 and Table 5, and Figure 17.

To illustrate the effectiveness of the adopted EKF scheme during static situation and tag tracking, we conduct the above experiments. The experimental results as shown in Figure 12, Figure 13, Figure 15 and Figure 16, illustrate that the RTLS designed in this paper has stable performance in tracking and has high positioning accuracy while ensuring positioning continuity. The RMSEs of the static situation are approximately 0.065 m and 0.072 m, and the RMSEs of tag tracking are approximately 0.192 m and 0.213 m. So, we can achieve a positioning error of less than 10 cm when the tag is fixed and less than 30 cm when the tag is moving. Limited by the hardware’s clock resolution, it is hard to completely eliminate clock bias and positioning errors.

### 7.6. Comparison of Positioning Performance of Different Schemes

Table 6 demonstrates the positioning performance of eleven schemes using the TDoA method. Atlas’s scheme and Bitcraze’s scheme are open source, and the other eight schemes are commercial. According to the data provided by each solution provider, the static positioning accuracy is about 10 cm, and the dynamic positioning accuracy is about 30 cm. Compared with our proposed scheme, the positioning accuracy is almost the same, because any mature solution uses unique algorithms to eliminate external errors, so the positioning accuracy depends on the performance of the UWB RF chip. The performance of our proposed scheme is consistent with the mainstream schemes, and it has flexible expansion in the aspect of multi-anchor cascade.

## 8. Conclusions and Future Work

This paper reviews the existing technologies and solutions for precise positioning, analyzes the advantages of UWB technology, and discusses the current available solutions of mainstream manufacturers in UWB. Then, we summarize the algorithms and solutions needed to build a real-time localization system based on UWB. To address the deficiencies and challenges of the existing solutions, a comprehensive UWB-based RTLS is proposed.

First of all, we design and implement the hardware and software of anchors and tags based on UWB, which have good performance and low power consumption. Then, we propose the new wireless clock synchronization (WCS) method and define the time-base selection strategy for TDoA algorithm in signal master anchor and multiple master anchors systems. Meanwhile, the EKF method for solving TDoA issues is introduced for non-linear dynamic systems, and it is useful in moving target tracking with the real-time positioning accuracy up to 30 cm. Finally, we discuss the relationship between anchor deployment and positioning accuracy.

At present, DW1000 chip follows the standard of IEEE 802.15.4-2011. Meanwhile, the IEEE 802.15.4z standard is announced to support DW3000, Apple U1, and NXP SR100T. The IEEE 802.15.4z defines new features based on the original standard, with enhanced security, lower power consumption, and longer transmission distance. We will continue our research based on the new standard, including TDoA, arrival of angle (AoA), and phase difference of arrival (PDoA).

## Figures and Tables

**Table 1:** Synchronization error of different WCS algorithms with respect to the synchronization period.

Sync-Error [ns]				
Synchronization Period (ms)	LI	PID	KF	LI-KF (Ours)
150	0.25	0.30	0.21	0.17
200	0.31	0.38	0.26	0.21
300	0.39	0.48	0.33	0.27
500	0.50	0.61	0.42	0.36

**Table 2:** The positions of the anchors.

Anchor Label	x_axis Coordinate (m)	y_axis Coordinate (m)
MA	0.00	0.00
SA2	6.00	0.00
SA3	6.00	4.00
SA4	0.00	4.00

**Table 3:** The positions of the anchors.

Anchor Label	x_axis Coordinate (m)	y_axis Coordinate (m)
MA1	0.00	5.25
SA2	0.00	0.00
SA3	5.05	0.00
SA4	11.02	5.25
SA5	11.02	9.88
MA6	5.05	9.88

**Table 4:** RMSE of static situation with respect to the different location estimation algorithms.

RMSE (cm)				
Number of Anchors	NI	SX	PF	EKF (Ours)
4	8.70	7.80	7.30	6.50
6	9.40	8.60	7.80	7.20

**Table 5:** RMSE of tag tracking with respect to the different location estimation algorithms.

RMSE [cm]				
Number of Anchors	NI	SX	PF	EKF (Ours)
4	25.50	24.10	21.30	19.20
6	28.20	26.30	22.90	21.30

**Table 6:** Positioning Performance of Different Schemes.

Scheme	Source	Static	Dynamic
SUSTechRTLS	**Lab Research**	**5–10 cm**	**about** **30 cm**
DecaWaveRTLS	Commercial	about 10 cm	about 30 cm
JINGWEI	Commercial	about 10 cm	10–30 cm
ATLAS	Open Source	about 10 cm	20–30 cm
woxuwireless	Commercial	about 10 cm	about 30 cm
Bitcraze	Open Source	about 10 cm	10–30 cm
EHIGH	Commercial	about 10 cm	about 30 cm
Sewio	Commercial	less 10 cm	about 30 cm
Localsense	Commercial	about 10 cm	10–30 cm
zebra	Commercial	about 10 cm	10–30 cm
ubitraq	Commercial	about 10 cm	10–30 cm

## Abbreviations

The following abbreviations are used in this paper:

UWB	Ultra-Wide Band
RTLS	Real-Time Localization System
RFID	Radio Frequency Identification
BLE	Bluetooth Low Energee
WiFi	Wireless Fidelity
FCC	Federal Communications Commission
WSN	Wireless Sensor Network
OFDM	Orthogonal Frequency Division Multiplexing
WCS	Wireless Clock Synchronization
U-WPN	UWB-based Wireless Positioning Network
TDoA	Time Difference of Arrival
ToA	Time of Arrival
ToF	Time of Flight
TWR	Two-Way Ranging
RSSI	Signal Strength Indication
CLE	Central Localization Engine
MA	Master Anchor
SA	Slave Anchor
CCP	Clock Calibration Packets
LI	Linear Interpolation
PID	Proportion Integration Differentiation
EKF	Extended Kalman Filter
UKF	Unscented Kalman Filter
PF	Particle Filter
DoP	Dilution of Precision
ML	Maximum Likelihood
SX	Spherical-intersection
NI	Newton Iteration
